# A distribution dependence study on the impacts of health rights’ accessibility on the overwork of migrant workers in China using quantile-on-quantile method

**DOI:** 10.3389/fpubh.2025.1634554

**Published:** 2025-08-07

**Authors:** Shengzhi Zhang, Yanlong Deng, Yu Zhang

**Affiliations:** ^1^School of Social and Public Administration, East China University of Science and Technology, Shanghai, China; ^2^School of Humanities and Public Administration, Shanghai Open University, Shanghai, China

**Keywords:** health rights’ accessibility, overwork, migrant worker, generational disparities, QQR

## Abstract

**Introduction:**

Overwork is a typical phenomenon in developing countries, especially in China, and also a significant issue that restricts the high-quality development of labor markets. Protecting the health rights’ accessibility of migrant workers in China, addressing persistent issues of overwork, and reducing generational disparities in well-being are urgent concerns. Investigating the formation mechanisms and generational variations in migrant workers’ overwork through the lens of health rights accessibility enriches theoretical explanations of overwork’s causes while offering actionable strategies for mitigation.

**Methods:**

Based on the China Migrants Dynamic Survey (CMDS), this study empirically tests the impact of health right’s accessibility on migrant workers’ overwork and its intergenerational differences using the quantile-on-quantile regression (QQR) and composition test method.

**Results:**

Health rights’ accessibility significantly reduces migrant workers’ overwork, with a 1-unit improvement correlating with a 4.22% decline in overwork rate and 2.36-h weekly reduction in overwork hours. The inhibitory effect is significantly stronger among new-generation migrants compared with first-generation counterparts. Threshold sensitivity exists: significant impacts emerge only when accessibility exceeds 0.4 for first-generation and 0.2 for new-generation migrants.

**Discussion:**

In about half of the sample cities, excessive health rights’ accessibility paradoxically increases migrants’ labor supply, revealing local policy failures.

## 1 Introduction

Migrant workers in China are defined as individuals whose household registration remains rural but who engage in non-agricultural employment in urban or other regions for 6 months or more. This population emerged as a distinct social group during the country’s rapid industrialization, urbanization, and reform and opening-up, and has since evolved into a defining and indispensable component of China’s new-type urbanization process (1). In recent years, the size of the migrant workers has continued to grow steadily.^1^ According to the China Ministry of Agriculture and Rural Affairs (2024), efforts to improve both the quantity and quality of migrant labor have supported this upward trend. Data from the National Bureau of Statistics in China show that the total number of migrant workers reached 298 million in 2023, an increase of 1.91 million (0.6%) from the previous year.^2^ Migrant workers play a vital role not only in driving China’s industrial growth and urban development, but also in fostering urban economic vitality, rural transformation, and national modernization (2). They supply a stable labor force that supports sustained economic expansion, facilitates the delivery of essential services, and accelerates the processes of industrialization and urban modernization. Simultaneously, they promote urban–rural integration by raising household incomes, improving living conditions in rural areas, and introducing new knowledge and technologies that contribute to industrial upgrading and rural revitalization (3). As such, protecting the accessibility of their health rights, addressing persistent issues of overwork, and reducing generational disparities in well-being are urgent concerns.

In China’s labor market, despite moderate wage growth in recent years, migrant workers continue to earn disproportionately low incomes. According to the 2023 National Migrant Worker Monitoring Survey, their average monthly wage reached 4,780 yuan—a 3.6% increase from the previous year. However, both the absolute income level and its rate of increase remain well below those of urban residents. In an effort to enhance their earnings and access more job opportunities, a substantial share of migrant workers engage in prolonged and intensive labor. In 2023, 64.4% of migrant workers exceeded the daily 8-h limit stipulated by China’s Labor Law; 84.4% surpassed the statutory 44-h workweek, averaging 9 h of work per day with fewer than four rest days per month. Alarmingly, 47% reported working year-round without a single day off. Such persistent overwork heightens the risk of chronic health conditions, including cardiovascular diseases, diabetes, and mental health disorders (4, 5), while reinforcing unhealthy work habits and diminishing overall well-being (6, 7). As the primary breadwinners in their households, migrant workers’ overwork not only endangers their physical and mental health—at times resulting in fatal outcomes such as death from overwork—but also exacerbates household economic vulnerability and the risk of poverty relapse. From a broader labor market perspective, widespread overwork disrupts supply–demand balance, undermines employment stability, and poses a barrier to achieving high-quality, inclusive, and sustainable development. As such, overwork-related health crises have shifted from a latent concern to a visible social issue in China, underscoring the urgent need for empirical research and targeted policy responses—particularly those addressing disparities in the accessibility of health rights.

Overwork among migrant workers in China stems from systemic factors such as technological advancement, industrial upgrading, labor market competition, and sector-specific demands (8). More than 56% are concentrated in manufacturing, construction, and retail—sectors characterized by extended working hours. As primary breadwinners, many are compelled to overwork to meet household expenses and cover out-of-pocket healthcare costs, a burden worsened by urban public health systems that prioritize registered residents. Health rights’ accessibility refers to the ability of workers to obtain necessary health protections, including medical services, work injury insurance, and occupational disease prevention. Limited accessibility to health rights forces migrant workers into a “delay minor illnesses, endure major ones” survival mode, ultimately diminishing productivity and undermining societal efficiency. Promoting health equity among migrant workers is therefore essential to alleviating overwork, yet it requires a nuanced understanding of the generational disparities within this population. Amid China’s rapid socioeconomic transformation, the migrant labor force has experienced a generational shift, marked by differing experiences and needs across cohorts. First-generation migrants (born before the 1980s), with lower educational attainment and unstable employment, often suffer chronic health issues while lacking insurance coverage and awareness of their rights (9). In contrast, new-generation migrants (born after the 1990s) express stronger demands for health equity but are still constrained by job insecurity, leading them to overwork for precautionary savings despite higher education levels and greater rights consciousness. Addressing overwork is thus vital not only for protecting migrant well-being but also for advancing sustainable urbanization. This study investigates how health rights’ accessibility influences overwork across generations, offering new insights to balance migrant welfare with labor market resilience through rights-based reforms.

## 2 Literature review and hypothesis

Overwork constitutes a central concern in studies on the labor conditions of migrant workers. Existing literature widely defines overwork as a labor pattern where individuals work beyond statutory or reasonable limits, leading to adverse physical and psychological health outcomes. The International Labor Organization characterizes working more than 48 h per week as overwork, warning that such conditions significantly elevate the risks of workplace injuries, occupational diseases, and mental health disorders. Under China’s Labor Law, statutory work hours should not exceed 44 h weekly, with labor beyond this threshold deemed excessive (10). However, migrant workers consistently face prolonged working hours: 64.4% exceed 8 h daily, and 84.4% of migrant workers in cities work over 44 h weekly, averaging 9 h daily and fewer than 4 rest days monthly. Such systemic overwork violates labor regulations and exacerbates health risks (11), reflecting a cycle where prolonged hours compound physical and psychological decline (7).

The overwork of migrant workers in China stems from a combination of socioeconomic, labor market, household, and individual-level factors (12, 13). Labor market competition is a primary driver (14), with rapid urbanization and economic expansion, the supply of rural migrant labor has surged, while low-skilled job opportunities remain limited. This imbalance pushes migrant workers to extend working hours to maintain employment or boost income (15). In construction and manufacturing, in particular, low wages and unstable jobs force workers to rely on excessive overtime for subsistence. Technological advancement and industrial upgrading have further intensified labor demands. Migrant workers must now adapt to both physical and technical requirements, often at the cost of rest time (16). Even in high-tech sectors, where conditions have improved, heightened performance expectations and time pressures contribute to increased workloads (17). Household financial burdens also play a significant role (18). As primary earners, many migrant workers work long hours to support housing payments, children’s education, and older adult care (13, 19), often sacrificing holidays and rest days. Individual characteristics further shape overwork patterns. Young, male, and less-educated migrant workers tend to have weaker bargaining power and are more susceptible to labor exploitation and extended work hours (20).

As a core labor right, Health rights’ accessibility directly affects productivity and quality of life (14, 21). For migrant workers in China, access is especially limited due to the hukou system and informal employment arrangements. Scholars typically measure health rights’ accessibility across dimensions such as availability of medical resources, injury insurance coverage, and occupational health services. Medical resource access concerns not only basic public health services but also timely treatment when illness occurs (22). Injury insurance determines whether migrant workers can receive compensation and care after work-related incidents (23). Higher health rights’ accessibility is associated with better physical health and work efficiency, as adequate protection reduces labor-related health risks and helps workers manage job demands more effectively (17). For migrant workers, insufficient coverage raises occupational health risks. Low injury insurance participation often leads to delayed treatment and lack of compensation (24), while unequal distribution of medical resources restricts access to urban-level healthcare. Ultimately, poor health access not only diminishes life quality but also reinforces overwork patterns among this group. Therefore, the paper puts forward the first theoretical hypothesis:

*H1*: The lack of health rights’ accessibility will further aggravate the overwork of migrant workers.

Generational disparities among China’s migrant workers reflect significant differences in attitudes toward overwork and demands for health rights’ accessibility, shaped by variations in social context, education, and values (25, 26). The first generation entered cities when social protection systems were weak. Under economic and family pressures, they often accepted long hours to secure income (27, 28). In contrast, the new generation, raised amid expanding social welfare (29), values work-life balance and is less willing to endure excessive working hours (30, 31). Generational gaps also exist in perceptions of health rights. With limited education, the older cohort tends to lack awareness and undervalue health protection, prioritizing income through extended labor. The new generation, being better educated, demands stronger health safeguards and prefers lawful means to defend their rights (32). Therefore, this paper proposes the second theoretical hypothesis:

*H2*: The response of overwork behavior of migrant workers from different generations to the accessibility of health rights shows heterogeneity.

## 3 Materials and methods

### 3.1 Data

Data on health rights’ accessibility and overwork among migrant workers are obtained from the 2018 China Migrants Dynamic Survey (CMDS), a large-scale, nationally representative survey conducted annually by China’s National Health Commission since 2009. The survey covers major inflow regions for migrant populations across 31 provinces (autonomous regions and municipalities) as well as the Xinjiang Production and Construction Corps. In 2018, the survey reached 1,459 county-level units, 3,776 township or subdistrict areas, and 8,993 neighborhood committees. With an annual sample size of approximately 200,000 households, the CMDS provides comprehensive information on migrant individuals and their families, including demographic characteristics, migration scope and patterns, employment status and social security coverage, income and housing, health conditions and access to basic public health services, as well as reproductive and family planning services. Additional control variables at the city level are sourced from the Easy Professional Superior (EPS) global statistical database platform.

### 3.2 Identification of overwork

This study identifies the overwork status of migrant workers from two dimensions: the extensive margin (whether the individual is overworked) and the intensive margin (the degree of overwork). For the extensive margin, a binary indicator is constructed, where a weekly working time above 44 h is coded as 1 (indicating overwork), and 0 otherwise. For the intensive margin, the severity of overwork is captured by the number of hours exceeding the 44-h threshold, calculated as the difference between actual weekly working hours and the legal standard.

Table 1 presents the descriptive statistics of the overwork rate and overwork hours among migrant workers in China. For the full sample, the average overwork rate is 74.23%, with an average of 16.24 h of excessive work per week. When disaggregated by generation, first-generation migrant workers exhibit a significantly higher overwork rate of 79.04% and average overwork hours of 18.95. In contrast, the new generation of migrant workers has a lower overwork rate of 71.28%, with an average of 14.54 excess hours per week. These findings reveal that overwork among migrant workers is a widespread issue in China, particularly among the first generation. Not only do they exhibit a higher likelihood of being overworked, but they also endure more severe overwork intensity—working, on average, 4.4 h more per week than their younger counterparts. This suggests that addressing overwork remains a substantial challenge, especially for the first generation of migrant workers.

**Table 1:** Descriptive statistics of overwork rate and overwork hours among migrant workers.

Variables	All sample	First-generation migrants	New-generation migrants
Overwork rate (%)	0.7423 (0.1277)	0.7904 (0.1140)	0.7128 (0.1383)
Overwork time (per week)	16.2414 (5.2680)	18.9486 (5.5268)	14.5352 (5.1790)
Sample size	152,000	60,344	91,643

Values represent means with standard deviations in parentheses.

**Figure 1:**
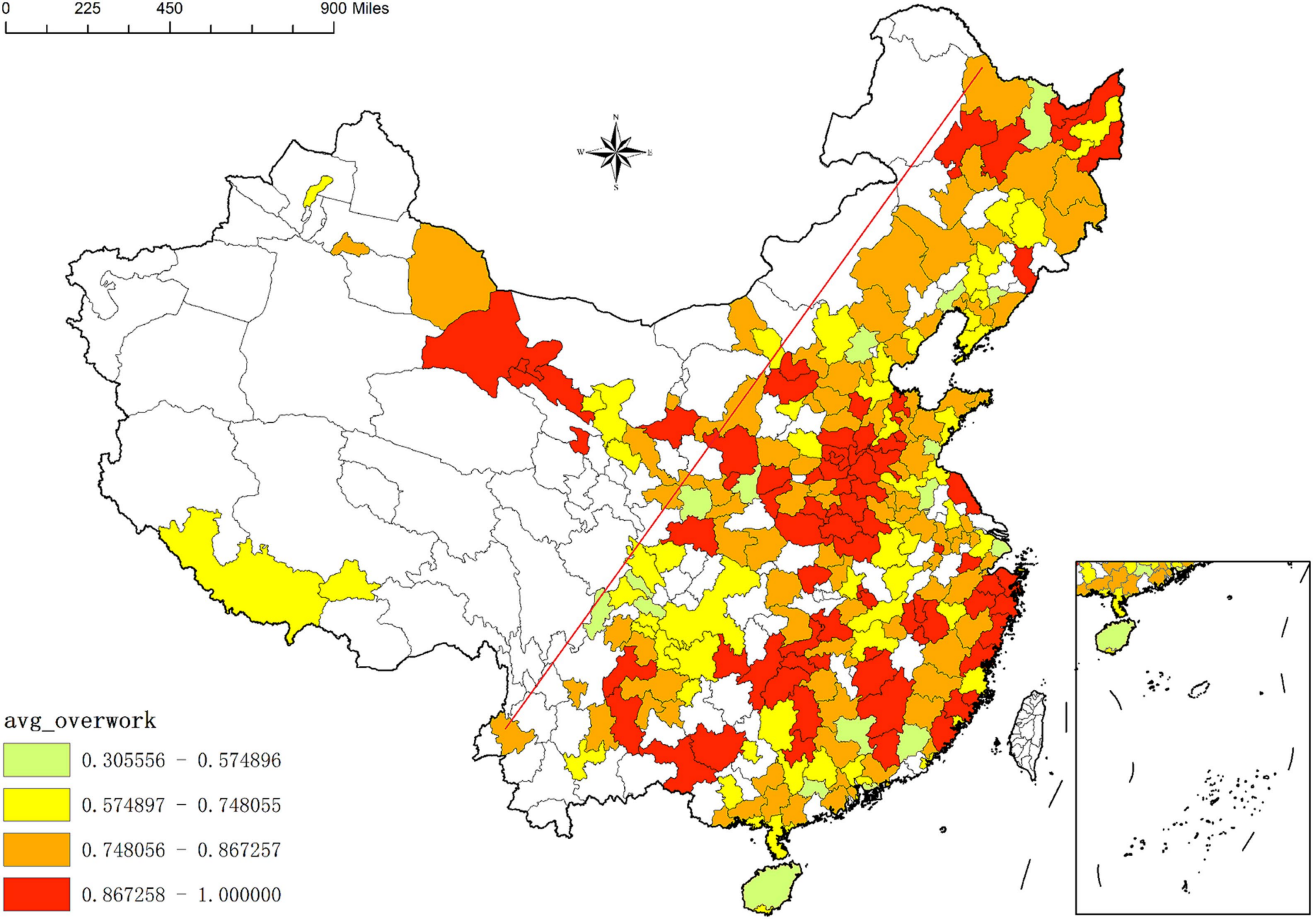
Overwork rate of internal migrant workers in different cities of China.

**Figure 2:**
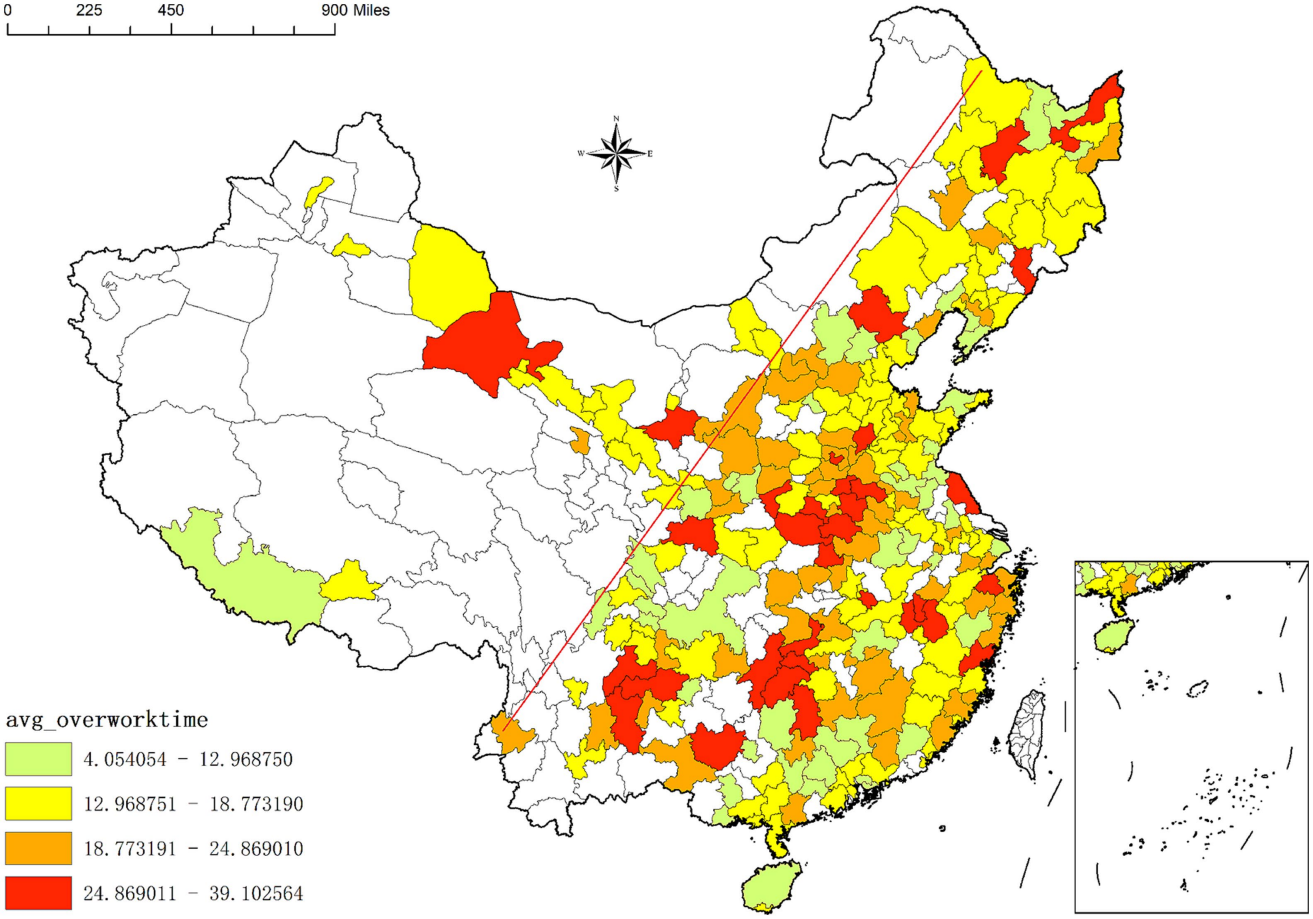
Overwork time of internal migrant workers in different cities of China.

Figures 1, 2 illustrate the spatial distribution of overwork rates and overwork hours among migrant workers across different cities in China. The blank area in the figures are the area not covered by the sample. Overall, migrant workers are predominantly concentrated in the eastern regions of the “Hu Huanyong Line,”^3^ a pattern consistent with the broader spatial distribution of population and economic development in China. However, cities with higher overwork rates and longer overwork hours are not necessarily those with the largest or densest migrant workers. For instance, cities with overwork rates exceeding 97% include Guoluo, Zhaotong, Zhoukou, Huaihua, Xiangxi, Dingxi, Tongchuan, Hanzhong, Heyuan, Longnan, Chongzuo, Shangluo, Diqing, Yingtan, and Laiwu. Similarly, cities where migrant workers experience the longest overwork hours—exceeding 31 h per week—include Diqing, Nujiang, Zhaotong, Guoluo, Shangluo, Kaifeng, Zhoukou, Qujing, Shangqiu, Hebi, Hanzhong, Jingdezhen, and Xiangxi. These findings suggest a geographical mismatch between the scale of migrant worker presence and the intensity of overwork, indicating that distributional disparities in health rights accessibility and labor conditions may be more severe in less economically developed regions.

### 3.3 Identification of health rights’ accessibility

The core explanatory variable in this study is the average level of health rights’ accessibility among urban migrant workers, which is measured across three dimensions: health records, health education, and medical insurance. Specifically, for the health records dimension, individuals who responded “a personal health record has been established locally” were assigned a value of 1; all other responses were coded as 0. For the health education dimension, individuals who reported having received any type of health education in their current community or workplace—such as on occupational disease prevention, infectious disease control, reproductive and maternal health, chronic disease prevention, mental health, or emergency self-help—were coded as 1; otherwise, 0. For the medical insurance dimension, individuals who indicated enrollment in any form of health insurance—such as the Urban and Rural Residents’ Basic Medical Insurance, Urban Residents’ Basic Medical Insurance, Urban Employees’ Medical Insurance, or publicly funded medical care—were assigned a value of 1; otherwise, 0. The three binary indicators were then summed to construct an individual-level health rights’ accessibility index. The city-level average of this index among all sampled migrant workers in each city was calculated and used as the final measure of health rights’ accessibility.

### 3.4 Model specification

#### 3.4.1 Benchmark regression model

The impact of health rights’ accessibility on the overwork of internal migrant workers has been supported by theoretical research. In order to further verify the response of the overwork of internal migrant workers in China to the accessibility of health rights in immigration city, this paper firstly constructs the following Probit econometric mode:


(1) $$\mathrm{pr}(\text{overwor}k_{\mathrm{ij}}=1)=\phi (\alpha +\text{βaccessibilit}y_{\mathrm{ij}}+\gamma_{1}M_{i}+\gamma_{2}Z_{j})$$

Where, the dependent variables overwork is a dummy variable for overwork of internal migrant workers *i in* city *j.* The independent variable accessibility represents the accessibility of health rights for internal migrant workers. β is the net effect of health rights’ accessibility on the overwork rate of internal migrant workers. M and Z are used to control the individual attribute factors and urban factors that affect the overwork of internal migrant workers.

In the control variables, individual attributes include age, age squared term, gender, marital status, educational attainment, health status, number of children, dummy of son, and individual income. Marital status is a dummy variable, coded as 1 for “first marriage” and “remarriage,” and 0 for “unmarried,” “cohabitation,” “divorced,” and “widowed.” Educational attainment is quantified using a Likert scale, assigning values 1–7 to “no formal education,” “primary school,” “junior high school,” “senior high school/technical secondary school,” “college,” “undergraduate degree,” and “postgraduate degree” based on ascending educational levels. Health status is measured by self-rated health outcomes, with “healthy,” “generally healthy,” “unhealthy but self-sufficient,” and “disabled” assigned values 4, 3, 2, and 1, respectively. In addition, following the practice of Wu et al. (33) and Wei (34), the number of children and the dummy variable of having a son are controlled in the regression equation. City-level attributes encompass economic development level (measured by per capita GDP), average wage, industrial structure (share of secondary and tertiary industries), foreign trade dependency (total import/export value as a percentage of GDP), and government public expenditure (scale of local fiscal spending). All raw data for control variables were obtained from the *China City Statistical Yearbook* for corresponding years, with missing values supplemented by municipal statistical yearbooks.

The Probit model effectively identifies the impact of health right’s accessibility on migrant workers’ overwork rates (i.e., the extensive overwork). To further examine whether health right’s accessibility significantly influences overwork duration (i.e., the intensive overwork), this section substitutes the binary overwork variable with a continuous duration measure, constructing an extended econometric Model (6) to analyze the relationship between health right’s accessibility and overtime labor supply. Additionally, given the divergent behavioral orientations and healthcare access disparities between new-generation and first-generation migrant workers, the analysis incorporates generational stratification to disentangle heterogeneous effects of health right’s accessibility on overwork across cohorts.


(2) $$\text{overwork}\_\mathrm{tim}e_{\mathrm{ij}}=\alpha +\text{βaccessibilit}y_{\mathrm{ij}}+\gamma_{1}M_{i}+\gamma_{2}Z_{j}+\varepsilon_{\mathrm{ij}}$$

#### 3.4.2 Quantile-on-quantile regression model

Quantile-on-quantile regression (QQR) is among the novel econometric models for analyzing the dependencies between two variables at different levels of quantiles. To study the effects of oil price on stock return, QQR model was developed by Sim and Zhou (35) by combining the traditional quantile regression (QR) and nonparametric method. The QQR model makes up for the deficiency of traditional OLS regression in explaining the nonlinear relationship between variables, and has the advantage of considering different quantiles of the dependent variable in traditional QR. Moreover, it can effectively observe the local influence of different fractions of independent variables and capture the time-varying characteristics (36).

For ease of understanding, let us start with the θth quantile regression $Q_{y}(\theta |x)$ :


(3) $$Q_{y}(\theta |x)=\alpha^{\theta }+\beta^{\theta }\cdot x$$

For observation i , the quantile residue is defined as:


(4) $$u_{i}=y_{i}-Q_{y}(\theta |x_{i})$$

Then, $\alpha^{\theta }$ and $\beta^{\theta }$ will be estimated by minimizing the expected loss:


(5) $$\begin{array}{c} \min_{\alpha^{\theta },\beta^{\theta }}Ε[\rho_{\theta }(u)]=Ε[u\cdot (\theta -\prod (u<0))]\\ =(\theta -1)\int_{-\infty }^{y^{\theta }}(y-Q_{y}(\theta |x))\mathrm{dF}(y)+\theta \int_{y^{\theta }}^{+\infty }(y-Q_{y}(\theta |x))\mathrm{dF}(y) \end{array}$$

where $\rho_{\theta }(u)$ is the tilted absolute value function, and $y^{\theta }$ is the θ th of dependent variable. The unbiased estimates of $\alpha^{\theta }$ and $\beta^{\theta }$ are given by:


(6) $$\begin{array}{l} \alpha^{\theta },\beta^{\theta }=\\ \underset{\alpha^{\theta },\beta^{\theta }}{\text{argmin}}[\sum_{i:y_{i}<y^{\theta }}(\theta -1)(y_{i}-Q_{y}(\theta |x_{i}))+\sum_{i:y\geq y^{\theta }}\theta (y-Q_{y}(\theta |x_{i}))] \end{array}$$

Define K(⋅) as a kernel function and let us turn to the estimation of quantile-on-quantile regression:


(7) $$\min_{\alpha^{\theta \eta },\beta^{\theta \eta }}\sum_{i=i}^{n}w_{i}(x^{\eta })\cdot \rho_{\theta }(y_{i}-\alpha^{\theta \eta }-\beta^{\theta \eta }\cdot (x_{i}-x^{\eta }))$$

where $w_{i}(x^{\eta })=K(\frac{x_{i}-x^{\eta }}{h})$ is the weight of each observation. Thus, the QQR method can be regarded as a Taylor expansion of $Q_{y}(\theta |x_{i})$ in the neighborhood of $x^{\eta }$ :


(8) $$Q_{y}(\theta |x_{i})\approx Q_{y}(\theta |x^{\eta })+\frac{\partial Q_{y}(\theta |x^{\eta })}{\partial x}\cdot (x_{i}-x^{\eta })$$

## 4 Results

### 4.1 Benchmark regression results

#### 4.1.1 The net effects of health rights’ accessibility on the overwork of migrant workers

Columns (1)–(3) of Table 2 report the overall average effects and generational disparities in the impact of *health rights’ accessibility* on the *overwork rate* of migrant workers. In terms of the overall effect, the estimated coefficient of health rights’ accessibility is significantly negative, indicating that improved accessibility of health rights significantly reduces the likelihood of overwork at the extensive margin. Specifically, a one-level increase in health rights’ accessibility is associated with an approximate 4.22% reduction in the probability of overwork among migrant workers. With respect to generational differences, the suppressive effect of health rights’ accessibility on overwork rate is statistically significant for both the first-generation and the new-generation migrant workers. However, the magnitude of the effect is more pronounced for the new generation, with a reduction of approximately 4.96%, compared to 3% for the first generation. Columns (4)–(6) of Table 2 present the average effects and generational disparities in the impact of health rights’ accessibility on the intensive overwork, measured by *weekly overworking hours*. At the aggregate level, each one-level increase in health rights’ accessibility is associated with a reduction of approximately 2.36 h in weekly overwork time. Similarly, the generational comparison shows that the reduction in overwork intensity is stronger among the new-generation migrant workers. The estimated effect for the first generation is a decrease of 2.14 h, whereas for the new generation it is 2.48 h, suggesting a stronger responsiveness to health rights among younger cohorts.

**Table 2:** Baseline regression estimation results.

Variables	Dependent variable: *overwork_rate*			Dependent variable: *overwork_time*		
	All sample	First-generation migrant workers	New-generation migrant workers	All sample	First-generation migrant workers	New-generation migrant workers
	(1)	(2)	(3)	(4)	(5)	(6)
*accessibility*	−0.0422^***^	−0.0300^***^	−0.0496^***^	−2.3600^***^	−2.1443^***^	−2.4795^***^
	(0.0015)	(0.0023)	(0.0020)	(0.0569)	(0.0996)	(0.0684)
*age*	−0.0062^***^	0.0241^***^	−0.0041	−0.2094^***^	0.9144^***^	−0.1701
	(0.0009)	(0.0036)	(0.0032)	(0.0351)	(0.1576)	(0.1115)
age* age	0.0001^***^	−0.0003^***^	0.0000	0.0026^***^	−0.0088^***^	0.0007
	(0.0000)	(0.0000)	(0.0001)	(0.0004)	(0.0016)	(0.0019)
*gender*	0.0615^***^	0.0312^***^	0.0712^***^	1.4292^***^	0.5780^***^	1.7507^***^
	(0.0024)	(0.0039)	(0.0031)	(0.0919)	(0.1719)	(0.1074)
*marriage*	0.0082^*^	0.0416^***^	−0.0252^***^	0.6276^***^	2.3197^***^	−0.0251
	(0.0041)	(0.0081)	(0.0053)	(0.1566)	(0.3543)	(0.1828)
*education*	−0.1042^***^	−0.0725^***^	−0.1130^***^	−3.6827^***^	−2.7934^***^	−3.9253^***^
	(0.0012)	(0.0021)	(0.0015)	(0.0457)	(0.0922)	(0.0528)
*health*	0.0081^*^	0.0115^*^	−0.0035	−0.7159^***^	−0.5672^**^	−0.9716^***^
	(0.0036)	(0.0045)	(0.0060)	(0.1386)	(0.1963)	(0.2064)
*children*	0.0252^***^	0.0111^***^	0.0380^***^	1.6254^***^	1.2258^***^	2.0851^***^
	(0.0023)	(0.0033)	(0.0032)	(0.0866)	(0.1437)	(0.1109)
*son*	0.0136^***^	0.0171^***^	0.0110^**^	0.3374^**^	0.4898^*^	0.2445^*^
	(0.0031)	(0.0046)	(0.0041)	(0.1173)	(0.2000)	(0.1432)
*lnincome*	0.0140^***^	0.0125^***^	0.0137^***^	1.8899^***^	1.1672^***^	2.2992^***^
	(0.0024)	(0.0037)	(0.0031)	(0.0897)	(0.1608)	(0.1064)
*lnrjgdp*	−0.0210^***^	−0.0154^***^	−0.0230^***^	−0.9753^***^	−0.9998^***^	−0.9251^***^
	(0.0027)	(0.0042)	(0.0036)	(0.1041)	(0.1850)	(0.1236)
*lnwage*	−0.2641^***^	−0.2799^***^	−0.2482^***^	−8.8084^***^	−10.4480^***^	−7.7020^***^
	(0.0086)	(0.0132)	(0.0113)	(0.3274)	(0.5784)	(0.3906)
*industry*	0.2274^***^	0.1733^***^	0.2602^***^	9.0854^***^	11.2189^***^	7.6714^***^
	(0.0339)	(0.0500)	(0.0455)	(1.2902)	(2.1942)	(1.5778)
*lnopen*	0.0039^***^	0.0071^***^	0.0061^***^	−0.4276^***^	−0.3438^***^	−0.4708^***^
	(0.0011)	(0.0016)	(0.0014)	(0.0405)	(0.0714)	(0.0484)
*lnexpenditure*	−0.0154^***^	−0.0112^**^	−0.0185^***^	0.6520^***^	0.7532^***^	0.5442^***^
	(0.0025)	(0.0038)	(0.0032)	(0.0933)	(0.1646)	(0.1115)
*_cons*	4.1800^***^	3.4051^***^	4.1324^***^	124.7659^***^	114.7581^***^	113.3157^***^
	(0.0830)	(0.1541)	(0.1162)	(3.1611)	(6.7625)	(4.0285)
F Stats.	1259.6204	227.8855	972.7268	1322.7813	217.1047	1047.7999
R^2^	0.1386	0.0719	0.1660	0.1445	0.0688	0.1765
Obs.	117,457	44,117	73,340	117,457	44,117	73,340

***p < 0.01, **p < 0.05, *p < 0.1, robust standard errors in parentheses (the same below).

Overall, health rights’ accessibility reduces *migrant workers’ overwork* at both the extensive and intensive margins. Greater access to healthcare services and occupational health protections lowers their need to work excessively for precautionary savings. This effect is more pronounced among new-generation migrant workers, who are more responsive to improvements in health rights. Compared to the first generation, they face less economic pressure and family responsibility, are less tolerant of overwork, and place more value on work–life balance. In contrast, first-generation workers are more willing to endure long hours due to stronger economic burdens, making them less sensitive to health entitlements.

#### 4.1.2 The net effects of individual attribute factors on the overwork of migrant workers

According to the estimation results of individual attributes, the impact of age on both the overwork rate and overwork hours of the overall sample of migrant workers exhibits a U-shaped trend (Table 2). This suggests that at early stages, both overwork rate and intensity decline with age, but start to increase again after a threshold age of approximately 31 for overwork rate and 40 for overwork hours. In contrast, among first-generation migrant workers, age shows an inverted U-shaped relationship with overwork: for overwork rate, nearly 90% of sample points lie to the right of the turning point, indicating that their overwork rate tends to decline with age. However, for overwork hours, about 75% of the sample falls to the left of the turning point, suggesting an increasing trend in overwork duration as they age. This implies that while first-generation workers may be less likely to overwork, those who do tend to work longer hours. In comparison, the effect of age on overwork rate and overwork hours among new-generation migrant workers is statistically insignificant, indicating that their overwork behavior is not influenced by age.

The estimated coefficients for gender, individual income, number of children, and dummy of son are all significantly positive across all regression models. This indicates that male migrant workers, those with higher individual income, more children, or at least one son tend to experience both higher overwork rates and longer overwork hours. These effects hold consistently for both first-generation and new-generation migrant workers. The coefficient for marriage is significantly positive in the full sample and in the first-generation group, but significantly negative among new-generation workers. This suggests that being married is associated with higher overwork rates and overwork hours among all migrant workers and the first generation specifically. However, for the new generation, being married reduces the probability of overwork, while it shows no significant effect on overwork hours. The coefficient for education is significantly negative in all models, implying that higher educational attainment effectively mitigates both the likelihood and intensity of overwork among migrant workers. As for health, better health status slightly increases overwork rates in the full sample and the first-generation group, while marginally reducing overwork hours across all groups. Nevertheless, the magnitude of these effects is relatively small.

#### 4.1.3 The net effects of urban attribute factors on the overwork of migrant workers

In addition to individual-level factors, city-level characteristics also significantly affect the overwork behavior of migrant workers. As shown in Table 2, the estimated coefficients of lnrjgdp (city-level economic development) and lnwage (average urban wage) are significantly negative across all regression models. This indicates that higher levels of local economic development and average wages are associated with a substantial reduction in both the likelihood and intensity of overwork among migrant workers. Specifically, a 1% increase in lnrjgdp leads to an approximate 2% decrease in the average overwork rate and a reduction of about 1 h in average weekly overwork time. Similarly, a 1% increase in lnwage is associated with a 25–28% decline in the overwork rate and a decrease of roughly 8–10 h in weekly overwork time. Moreover, the mitigating effect of lnrjgdp is more pronounced in reducing the overwork rate of new-generation migrant workers and the overwork hours of first-generation migrant workers. By contrast, increases in lnwage exert a stronger suppressing effect on both the overwork rate and overwork hours of the first-generation group.

The estimated coefficients for the industry variable are significantly positive across all regression models, indicating that a greater concentration of secondary and tertiary industries in a city is associated with a higher likelihood and intensity of overwork among migrant workers. In other words, a more service-and manufacturing-oriented industrial structure tends to exacerbate overwork issues rather than alleviate them. The coefficient of lnopen (trade openness) is significantly positive in the overwork rate equation but significantly negative in the overwork hours equation. This suggests that increased trade dependence elevates the average overwork rate (extensive margin), yet reduces the average overwork time (intensive margin) among migrant workers. In contrast, the estimated coefficient for lnexpenditure (public expenditure) is significantly negative in the overwork rate equation and significantly positive in the overwork hours equation. This implies that while greater public spending can help reduce the proportion of migrant workers engaged in overwork, it may simultaneously increase the weekly overwork hours among those who do overwork.

### 4.2 Quantile-on-quantile regression estimation

We employed two types of contour maps to visualize QQR coefficient estimates: one displaying raw values and the other showing filtered values based on significance levels (where insignificant coefficients are set to zero). The color mapping illustrates the direction and significance of coefficients: red indicates that health right’s accessibility significantly promotes overwork, blue denotes a significant inhibitory effect, and white areas represent statistically insignificant regions.

#### 4.2.1 QQR estimation of overwork rate

**Figure 3:**
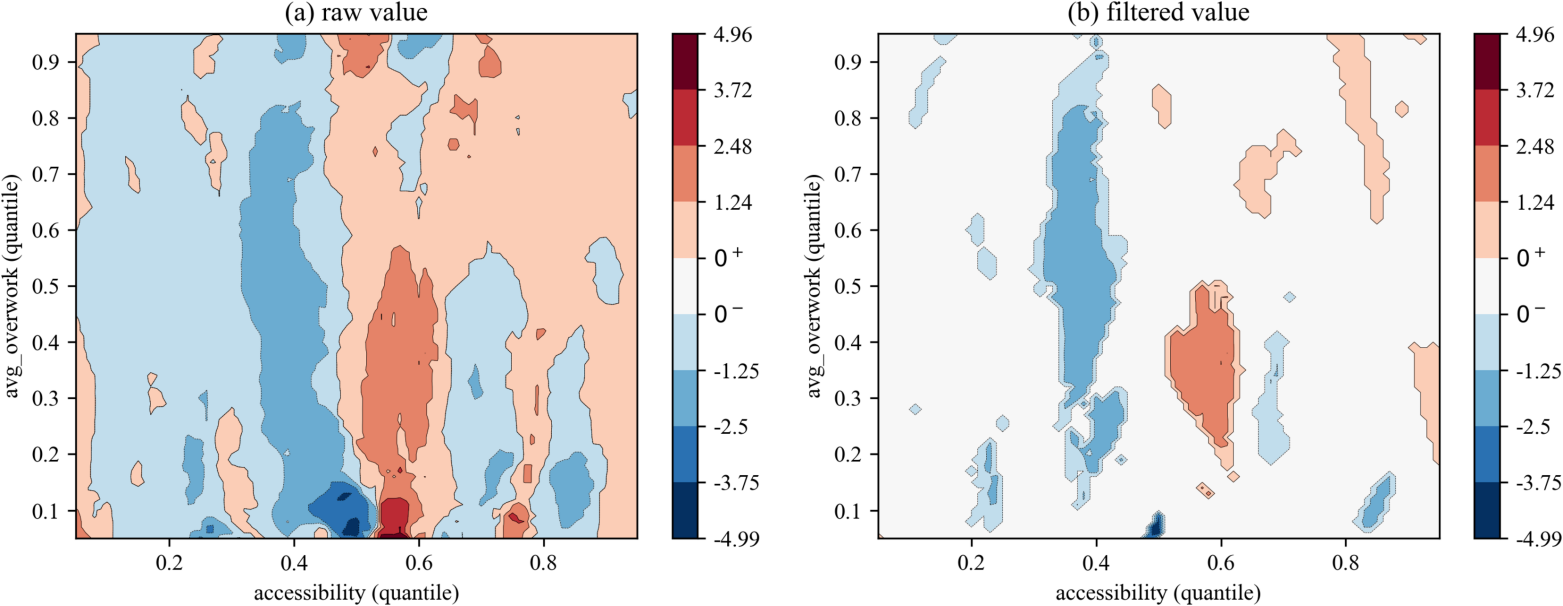
QQR estimation results of overwork rate (all).

The QQR contour plot based on the full sample (Figure 3) reveals a pronounced nonlinear distribution dependence in the marginal effects of health rights’ accessibility on the overwork rate of migrant workers. Specifically, the interaction between the quantiles of health rights’ accessibility and those of the overwork rate shows that when accessibility falls below the 0.3 quantile, its mitigating effect on overwork is largely insignificant. This suggests that low levels of health coverage are insufficient to meaningfully reduce excessive working hours. However, once health rights’ accessibility surpasses the 0.3 quantile threshold, the expansion of the blue region indicates a significantly stronger suppressive effect on the overwork rate. Yet, this alleviating effect diminishes at moderate to high levels of accessibility (above the 0.5 quantile). Notably, a structurally reversed pattern emerges: within the intersection of moderate health rights’ accessibility (0.5–0.6 quantiles) and low overwork quantiles (0.2–0.5), a significantly positive association is observed. This reversal may be attributed to a distorted income–leisure substitution effect. As broader health rights coverage is perceived by migrant workers as a form of implicit income, it may incentivize them to extend working hours in pursuit of higher consumption capacity. At the same time, enterprises tend to increase labor intensity due to the decline of health cost externalities, forming a transmission chain of “health equity improvement—labor tolerance improvement—active/passive extension of working hours.” In this sense, rigid institutional designs in health accessibility policies may inadvertently trigger a rebound in labor supply.

**Figure 4:**
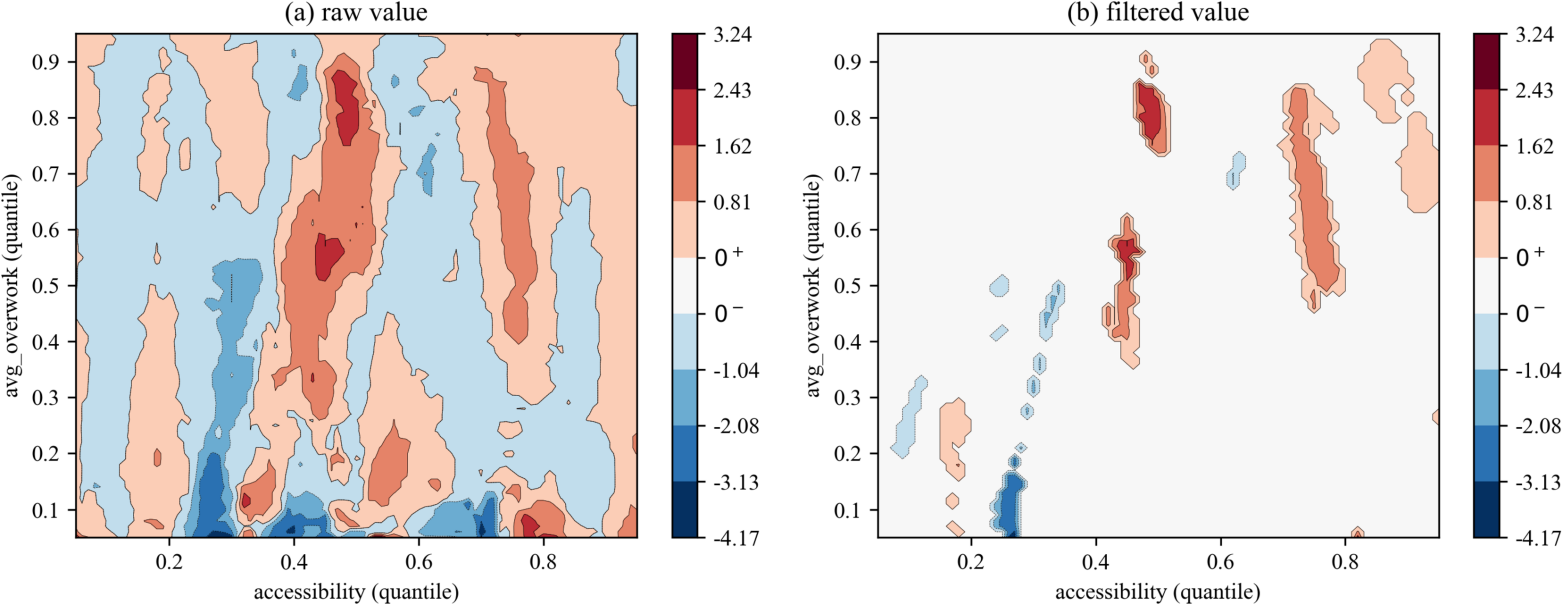
QQR estimation results of overwork rate (first-generation).

The sub-sample regression results reveal a clear intergenerational divergence in the distribution-dependent effects of health rights’ accessibility on the overwork rate of migrant workers. The QQR contour plot for the first generation (Figure 4) exhibits a “low-sensitivity, low-threshold” pattern. On the one hand, the mitigating effect of health rights’ accessibility is only marginally significant within a narrow intersection of its 0.25–0.4 quantile range and low overwork quantiles (below 0.5). The effect intensity shows a monotonic decline along both axes, with the strongest suppressive impact observed at the 0.25–0.1 quantile combination, it means when both health rights’ accessibility and overwork rate are relatively low (represented by the dark blue region). On the other hand, in the intersection of moderate-to-high health rights’ accessibility and high overwork quantiles (both above 0.45), health rights’ accessibility exhibits a localized but significant positive effect on overwork among older migrant workers. This suggests that, due to greater financial pressures and heavier family responsibilities, first-generation migrant workers with already high overwork rates may be incentivized to further increase their labor supply even when receiving only modest improvements in health rights coverage—seeking greater economic returns in exchange for more working hours.

**Figure 5:**
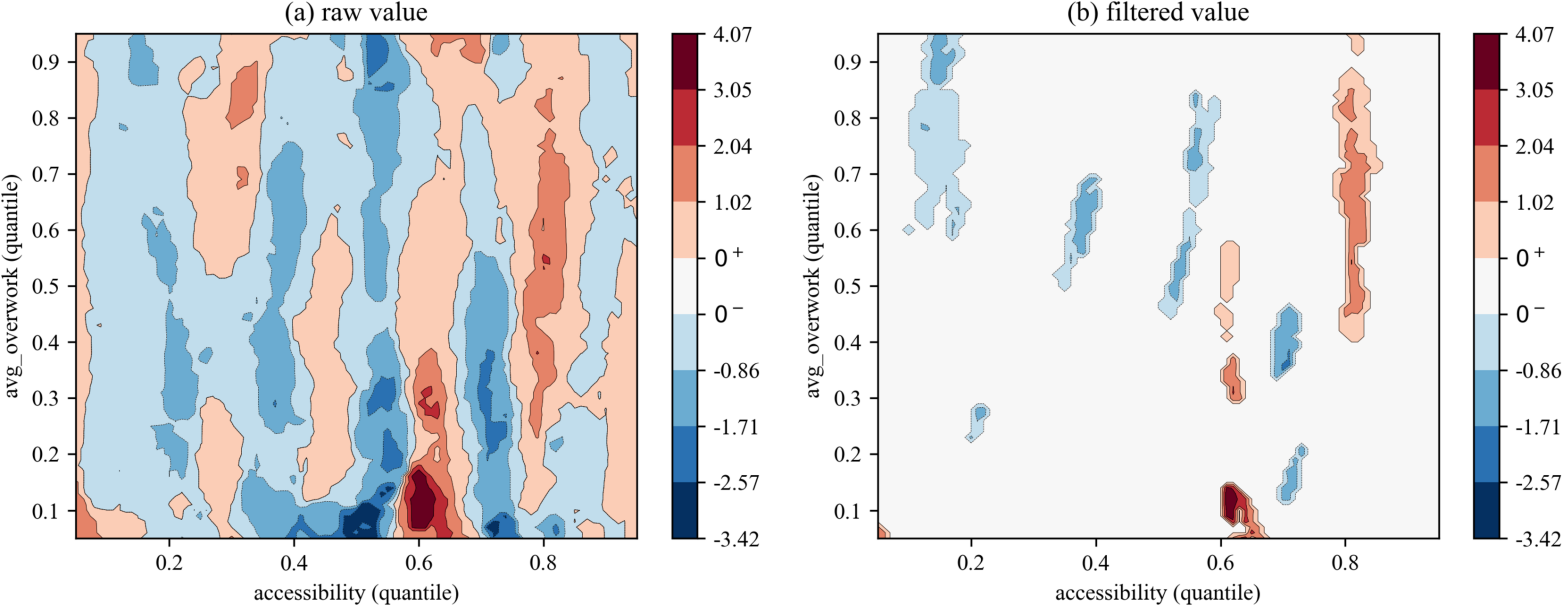
QQR estimation results of overwork rate (new-generation).

The QQR contour plot for the new generation of migrant workers (Figure 5) exhibits a “high-sensitivity, high-threshold” pattern. The mitigating effect of health rights’ accessibility is activated at a lower threshold—below the 0.2 quantile—and the extensive spread of blue areas indicates that its suppressive impact on higher overwork rate quantiles (above 0.6) is notably stronger than that observed among the first generation. Additionally, red regions appear earlier in the distribution space (with both axes above the 0.05 quantile), suggesting a more responsive distributional structure. However, in the upper quantiles of health rights’ accessibility (above 0.6), its effect on overwork becomes heterogeneous, alternating between negative and positive impacts. Specifically, significant positive effects are observed at the 0.6 and 0.8 quantiles, corresponding, respectively, to low (below 0.6) and high (above 0.4) overwork rate quantiles, forming distinct red bands across the contour map. A similar localized positive impact appears at the 0.7 quantile against lower overwork levels (below 0.5). This alternating pattern may be attributed to the unique human capital traits of the new generation. Their higher educational attainment enables them to better leverage health-related rights to reduce occupational risks. At the same time, improved access to social security may also incentivize them to voluntarily engage in more intensive labor in pursuit of higher earnings—thus potentially leading to a hidden crowding-out effect that offsets the original policy intention.

#### 4.2.2 QQR estimation of overwork time

**Figure 6:**
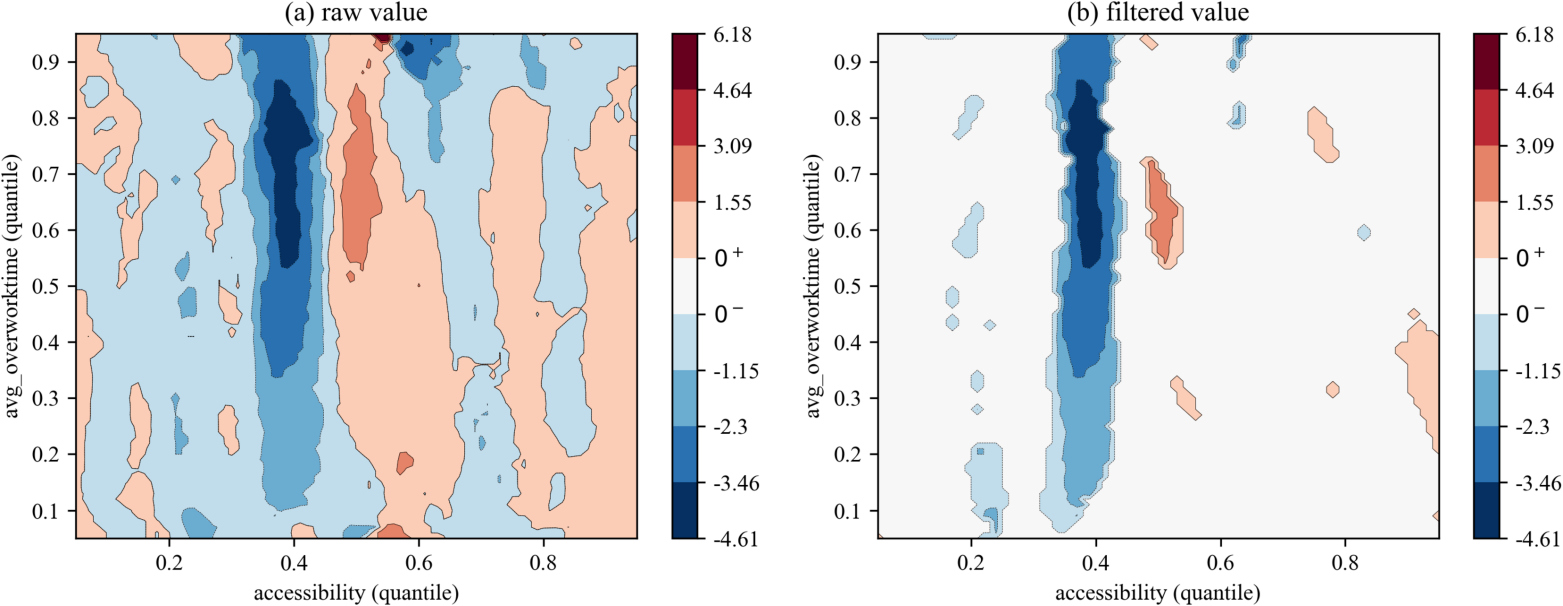
QQR estimation results of overwork time (all).

**Figure 7:**
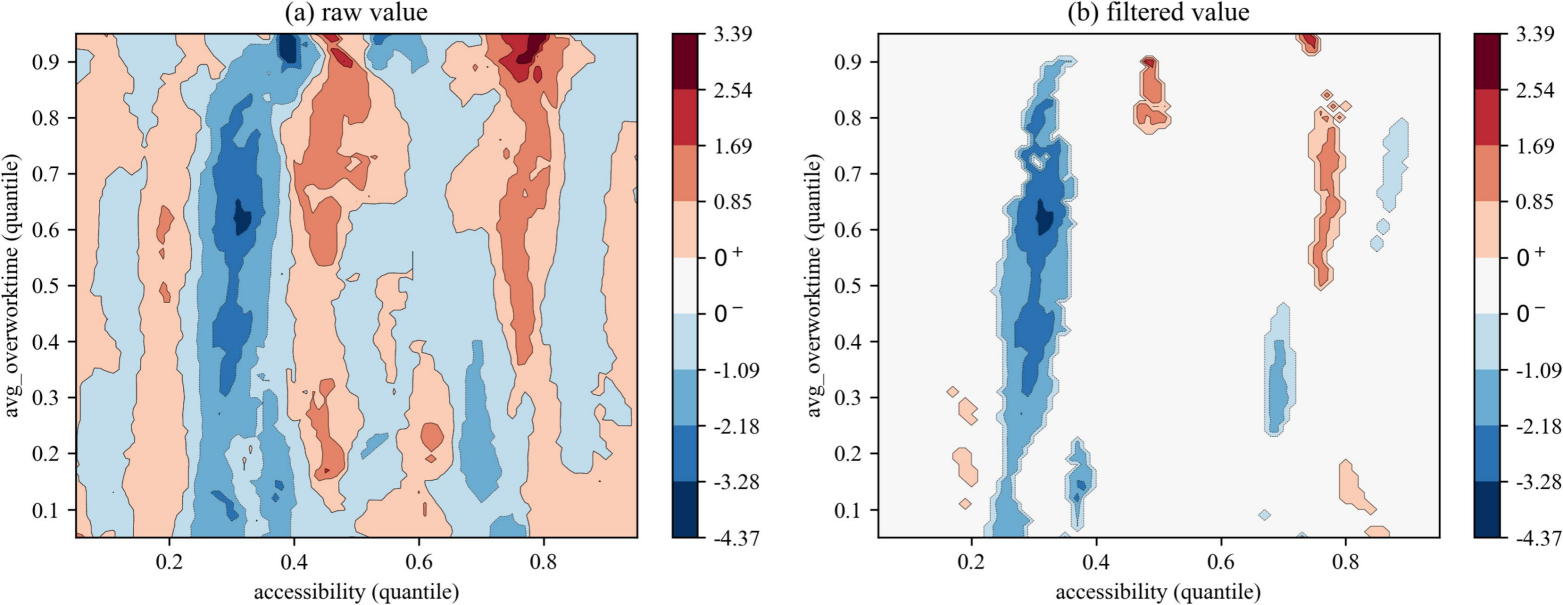
QQR estimation results of overwork time (first-generation).

**Figure 8:**
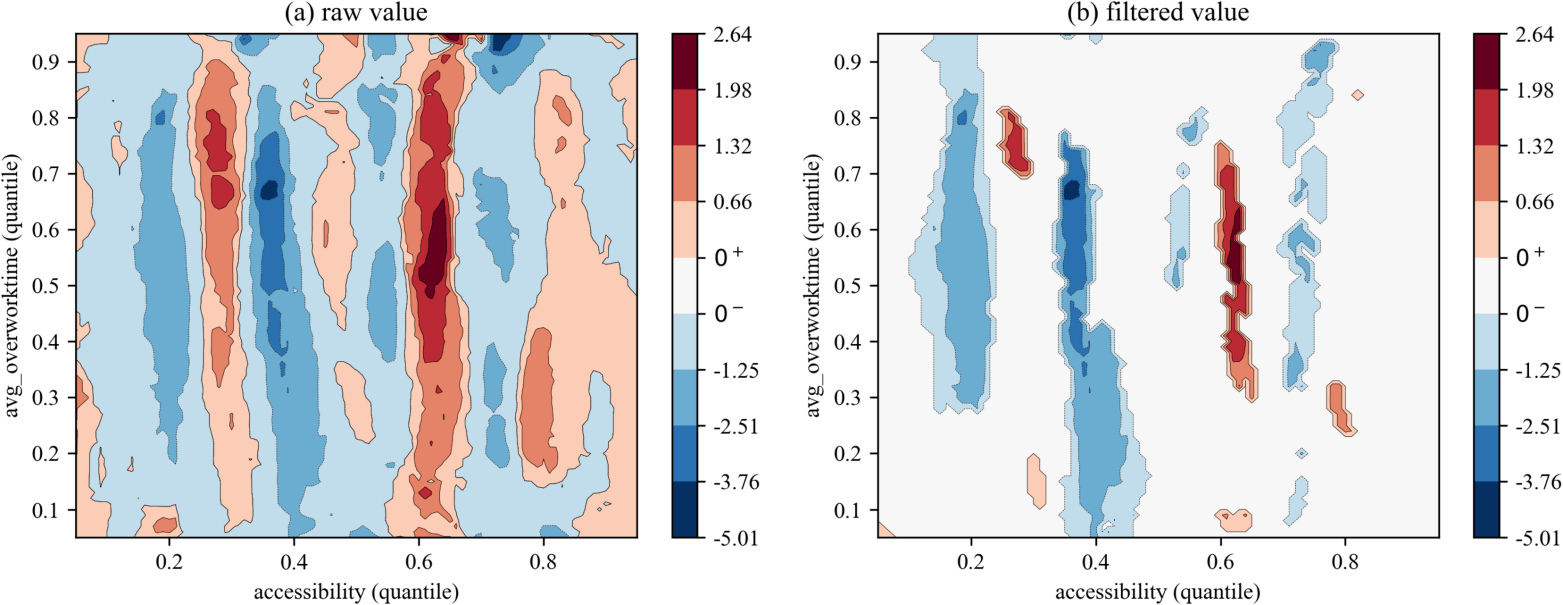
QQR estimation results of overwork time (new-generation).

The QQR analysis of the effects of health rights’ accessibility on the overwork duration of migrant workers (Figures 6–8) reveals a markedly different nonlinear distribution dependence pattern compared to that observed for overwork rate. From a vertical comparison across groups, health rights’ accessibility exhibits a more substantial and consistent mitigating effect on overwork duration than on overwork rate. In contrast, a horizontal comparison across Figures 6–8 uncovers an increasingly prominent alternation between negative and positive effects, reflecting a more complex interaction structure in the quantile space. This indicates that the impact of health rights accessibility on excessive working hours is not only stronger, but also more heterogeneous across the conditional distributions of both variables.

Specifically, the interaction between the quantiles of health rights’ accessibility and overwork duration for the full sample (Figure 6) reveals a distinct pattern of nonlinear distribution dependence. When health rights’ accessibility falls below the 0.40 quantile, its effect on overwork duration is generally negative, indicating a mitigating influence. Critically, within the 0.35–0.45 quantile range of health rights’ accessibility, a significantly negative effect is observed across nearly all quantiles of overwork duration, with the strongest suppression occurring at medium-to-high levels of overwork duration (between the 0.55 and 0.85 quantiles), as indicated by the deep blue regions. However, once health rights’ accessibility exceeds the 0.45 quantile threshold, its mitigating effect quickly diminishes and gives way to an alternating pattern of negative and positive impacts. This shift further supports the structural distortion associated with the “income–leisure substitution effect,” whereby improved access to health entitlements may paradoxically incentivize longer working hours through perceived income augmentation or employer-driven intensification of labor.

The subsample regression results further reveal a clear intergenerational divergence in the impact of health rights’ accessibility on the overwork duration of migrant workers. First, regarding the distributional patterns in the QQR contour plots, Figures 7, 8 exhibit distinct configurations from those based on overwork rate. Specifically, the first-generation and new-generation migrant workers demonstrate a “low-sensitivity–low-threshold” pattern and a “high-sensitivity-low-threshold” pattern, respectively. In other words, the overwork duration of new-generation migrant workers responds more sensitively to changes in health rights’ accessibility, and the alternating “negative–positive” effect emerges at lower quantiles of accessibility compared to their predecessors. Second, in terms of the effectiveness of the mitigating role of health rights’ accessibility on overwork duration, the new-generation group experiences significant suppression at several distinct quantile intervals—namely, at the 0.10–0.25, 0.35–0.45, and 0.70–0.75 quantiles of health rights’ accessibility. This multi-peaked distribution of inhibitory effects suggests that the working-hour adjustment function of health rights’ accessibility exhibits “phase-dependent effectiveness,” with the phenomenon being particularly pronounced among the new-generation migrant workers. A plausible explanation lies in their higher educational attainment and stronger health awareness, which enable them to more effectively recognize the potential benefits of health entitlements—such as early prevention of occupational diseases—and thereby reduce reliance on excessive working hours by minimizing health risk premiums.

### 4.3 Composition test for QQR

**Figure 9:**
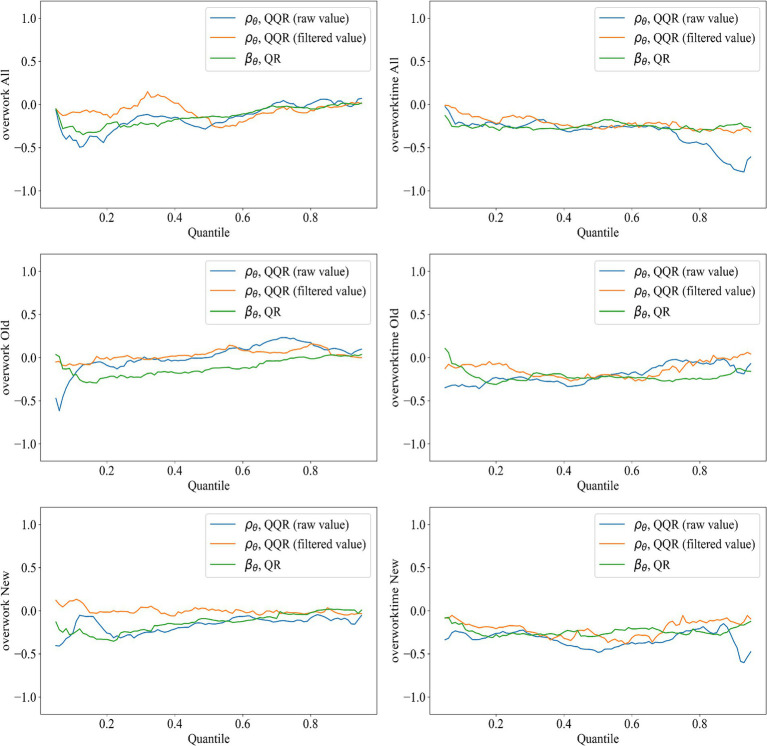
Composition test for QQR in each group.

Figure 9 compares the coefficients derived from the quantile regression (QR) with the average coefficients obtained from the two types of quantile-on-quantile regression (QQR) contour plots. The discrepancy between the two tends to diminish as the sample coverage becomes more comprehensive. With respect to overwork rate, in the full sample group, the filtered values exhibit a persistent positive bias, indicating that in the extreme ranges of health rights’ accessibility—specifically below the 10th percentile or above the 90th percentile—the marginal effect of accessibility on migrant workers’ overwork rate is negative. However, it is worth noting that when the overwork rate lies between the 50th and 80th percentiles, the filtered value bias becomes negative and gradually decreases with higher overwork percentiles. This suggests that under moderate levels of overwork rate, extreme values of health rights’ accessibility may exert a positive influence on the overwork rate. Subsample results further reveal that, in both the older and younger migrant cohorts, the filtered value bias remains positive and diminishes as the overwork quantile increases. This implies that in the extreme quantiles of health rights’ accessibility, the effect on overwork rate is generally negative and weakens as accessibility improves.

In contrast, for overwork duration, the bias of filtered values in the full sample group is notably smaller than that observed for overwork rate, suggesting a higher degree of consistency and robustness between the QQR and QR results when measuring the effect of health rights’ accessibility on working hours. From a generational perspective, the older migrant worker group exhibits near-zero bias in filtered values when overwork duration falls between the 30th and 60th percentiles. In other quantile ranges, however, a positive bias is observed, indicating that extreme values of health rights’ accessibility negatively impact overwork duration when working hours are either excessively low (below the 30th percentile) or excessively high (above the 60th percentile). The younger migrant cohort displays a pattern similar to the full sample, characterized by minor fluctuations in bias across quantiles. Across all groups, the overall bias in the filtered values is smaller than that of the original values. This supports the conclusion that adopting a filtering strategy—setting statistically insignificant coefficients to zero—can effectively mitigate the side effect of decomposition, namely the problem of information loss (36).

### 4.4 Further discussion

The QQR framework was used to estimate the city-level effects of health rights’ accessibility on migrant workers’ overwork. In the contour plots, red indicates a promoting effect and blue indicates a suppressing effect. Statistically significant effects are marked with an asterisk (*). By mapping observed city-level values of health rights’ accessibility and overwork outcomes, we matched QQR coefficients with individual sample cities (Supplementary Appendix), thereby revealing spatial heterogeneity in the distribution-dependent impacts and their underlying mechanisms.

Full-sample results show that among the 18 cities where health rights’ accessibility significantly increases overwork rates, most are underdeveloped inland or resource-based cities (e.g., Jiuquan), with only a few being provincial capitals (e.g., Chengdu, Wuhan). This spatial divergence can be explained from two angles. First, more efficient public service provision in eastern regions enhances the “health capital accumulation” effect, as better insurance systems reduce workers’ reliance on excessive hours. Second, in less developed cities with labor-intensive industries like construction and mining, expanded health coverage may reduce employers’ perceived occupational risk costs, inadvertently encouraging longer working hours. These findings are consistent with those for overwork duration.

Subsample results further support these patterns. Cities where health rights’ accessibility significantly promotes overwork—across both older and younger migrant cohorts—are mostly located in inland or traditional manufacturing regions (e.g., Dingxi, Jiangmen, Yueyang), likely reflecting institutional limitations in occupational health protection. Notably, for the younger generation, some provincial capitals in central China (e.g., Changsha, Nanchang) also show significant promoting effects, suggesting that better-educated workers may actively trade improved health security for higher earnings, leading to a paradoxical intensification of overwork.

## 5 Conclusions and policy implications

Drawing on the 2018 CMDS, this study applies OLS and QQR to empirically test the nonlinear effect and its intergenerational differences of health rights’ accessibility on the overwork of migrant workers from extensive margin and intensive margin. Results show that improved health rights’ accessibility significantly reduces overwork among migrant workers, though this effect exhibits complex characteristics in distribution dependence and group heterogeneity dimensions. Full-sample OLS regression estimates show that each level of enhanced health rights’ accessibility reduces overwork rates by 4.22% and decreases working hours by 2.36 h. Subsample analysis further reveals generational differentiation patterns, where the new-generation migrant workers exhibit significantly stronger inhibitory effects compared to older generations, indicating that intergenerational human capital disparities moderate policy responses.

QQR results reveal nonlinear and asymmetric patterns. For overwork rate, the effect of health rights’ accessibility is limited at low quantiles, but becomes increasingly negative at higher quantiles, forming an inverted-U shape. In mid-level accessibility, the impact even reverses direction for lower overwork groups. For overwork hours, a significant suppressive effect appears between the 0.35–0.45 accessibility range for higher-hour quantiles, but weakens beyond that point. Notably, younger workers show broader suppression across quantiles, reflecting greater efficiency in using health services and more strategic endurance in labor. These findings suggest that health rights have “phase-specific” effects, with generational human capital shaping differentiated responses. Furthermore, spatial analysis indicates substantial regional variation. In about half of the sample cities, health rights’ accessibility is associated with increased overwork, revealing local policy failures. This pattern is linked to labor market segmentation, unequal public services, and industrial structure—particularly in underdeveloped and resource-based regions, where improved health access may inadvertently incentivize longer work hours.

Based on the above findings, this study proposes the following policy recommendations. Firstly, it is necessary to implement differentiated investment strategy in health rights’ accessibility. For first generations of migrant workers, efforts should prioritize overcoming coverage thresholds by expanding grassroots health service networks and mainstreaming occupational disease screening to enhance policy responsiveness. For the new generations, early intervention should be emphasized while avoiding the risk of reversal of effects under high levels of equity coverage. Secondly, an integrated governance mechanism linking health and labor policies should be established. In the medium level of health rights’ accessibility, it is necessary to resolve the rebound in labor supply through income compensation mechanisms. And a normalized system for compliance review of working hours should be established for groups with high rates of overwork. Thirdly, the spatial alignment of policy resource allocation should be further optimized. For regions with significant effects of health rights policies, such as the eastern region, a “health rights points system” can be piloted to link the supply of rights with the fulfillment of social responsibility by employing enterprises. But in areas with low policy efficiency such as the central and western regions, priority should be given to improving the infrastructure for safeguarding labor rights.

## Data Availability

The original contributions presented in the study are included in the article/Supplementary material, further inquiries can be directed to the corresponding author.

## Footnotes

^1^《*Enhancing the Quantity and Quality of Migrant Workers’ Employment*》, Ministry of Agriculture and Rural Affairs of the People’s Republic of China, May 10, 2024.

^2^ https://www.stats.gov.cn/sj/zxfb/202404/t20240430_1948783.html

^3^The “Hu Line” (originally termed the “Aigun-Tengchong Line”) is a demographic-geographic demarcation proposed by Hu Huanyong in his 1935 article *The Distribution of China’s Population*. Following subsequent place-name changes, it has been renamed the “Aihui-Tengchong Line” or “Heihe-Tengchong Line.” The red diagonal lines in Figures 2, 3 depict this boundary.
