# Supplementary material for: A distribution dependence study on the impacts of health rights’ accessibility on the overwork of migrant workers in China using quantile-on-quantile method
Source: Front Public Health. 2025 Aug 7;13:1634554. doi: 10.3389/fpubh.2025.1634554 (PMC12367735; doi:10.3389/fpubh.2025.1634554)
Supplement: Supplementary file 1 [file Supplementary_file_1.docx]

**Appendix:**


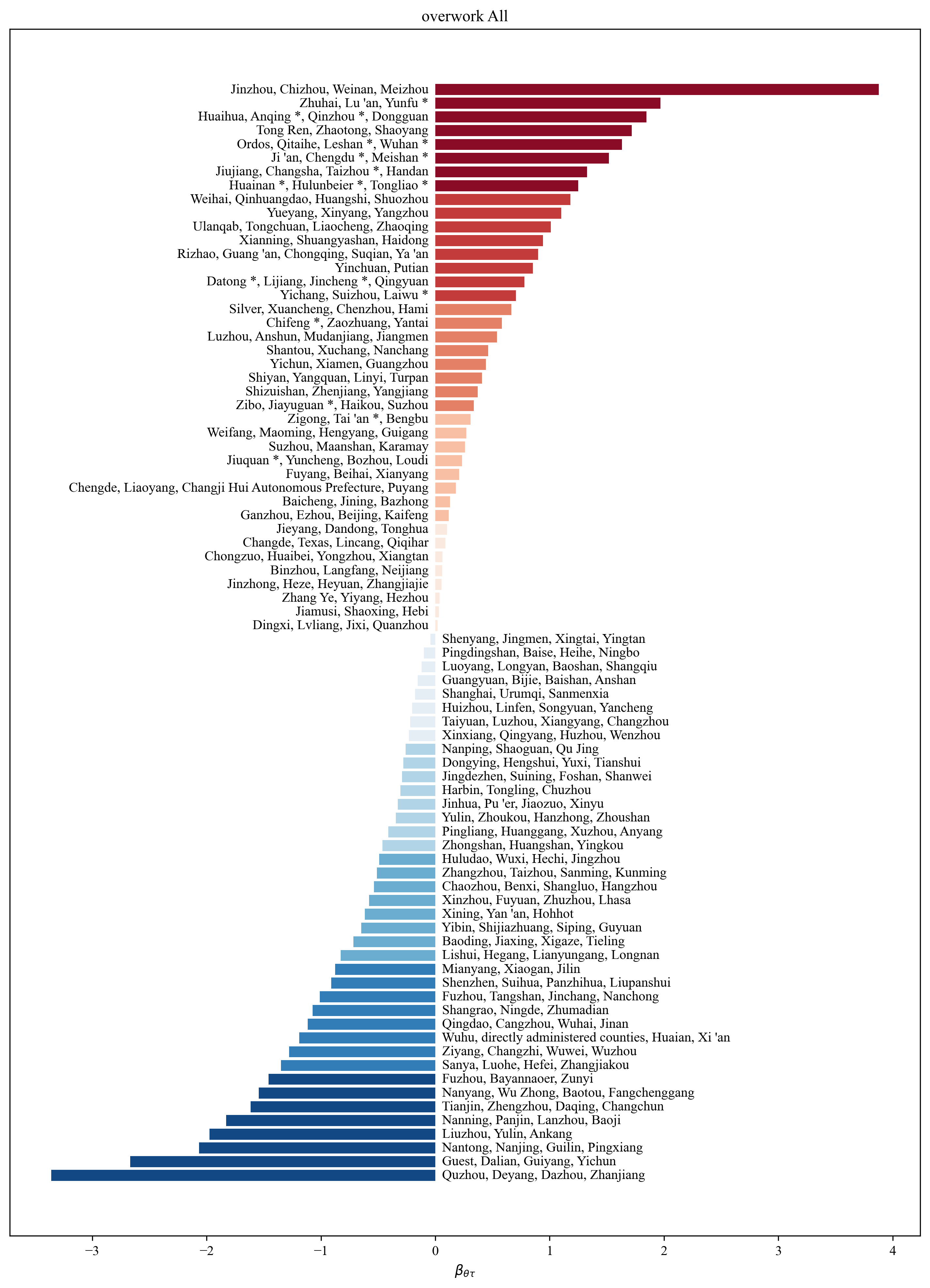


Fig. 1a. QQR estimates of effects of accessibility on overwork rate in each city (all)


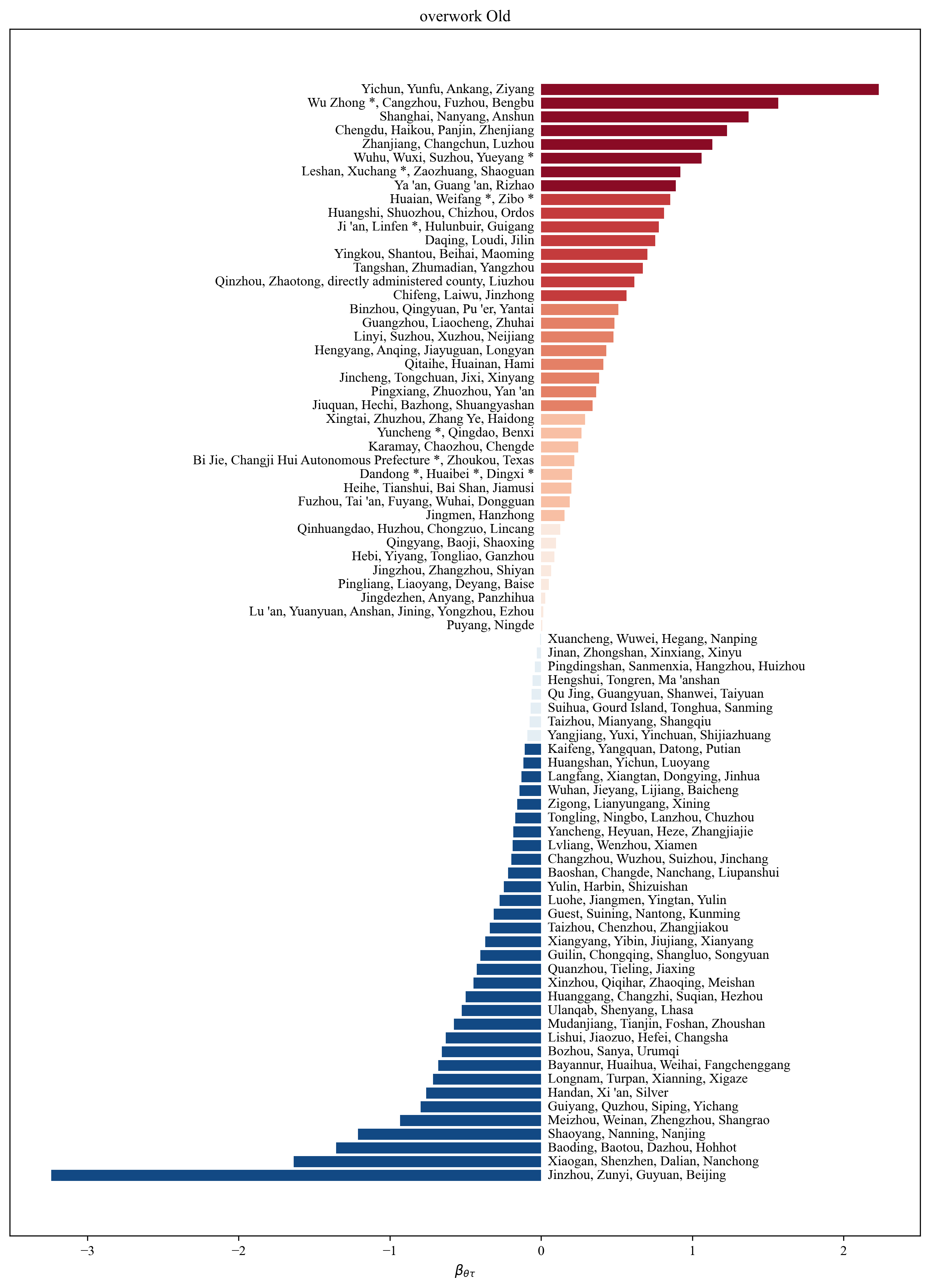


Fig. 1b. QQR estimates of effects of accessibility on overwork rate in each city (first-generation)


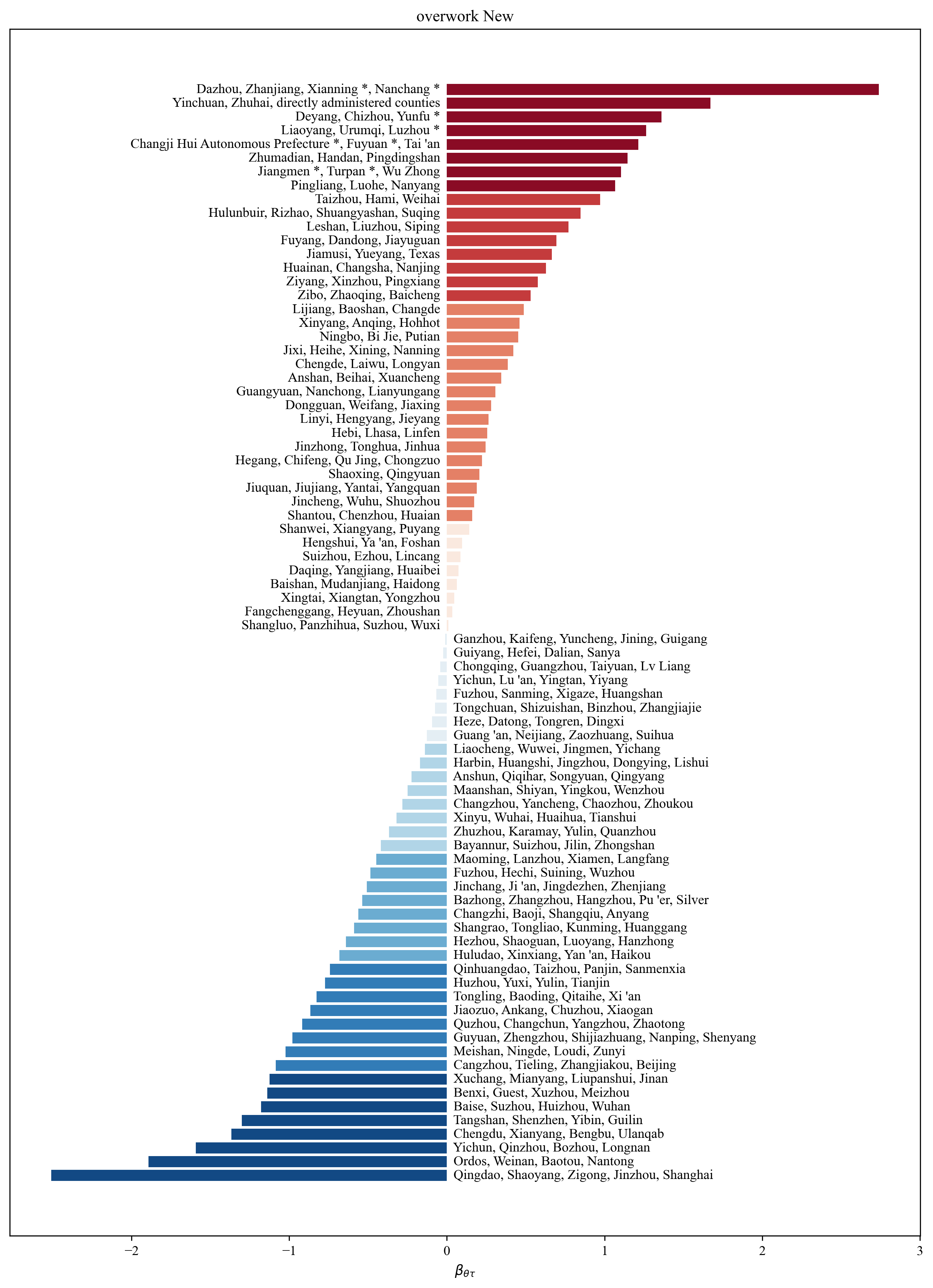


Fig. 1c. QQR estimates of effects of accessibility on overwork rate in each city (new-generation)


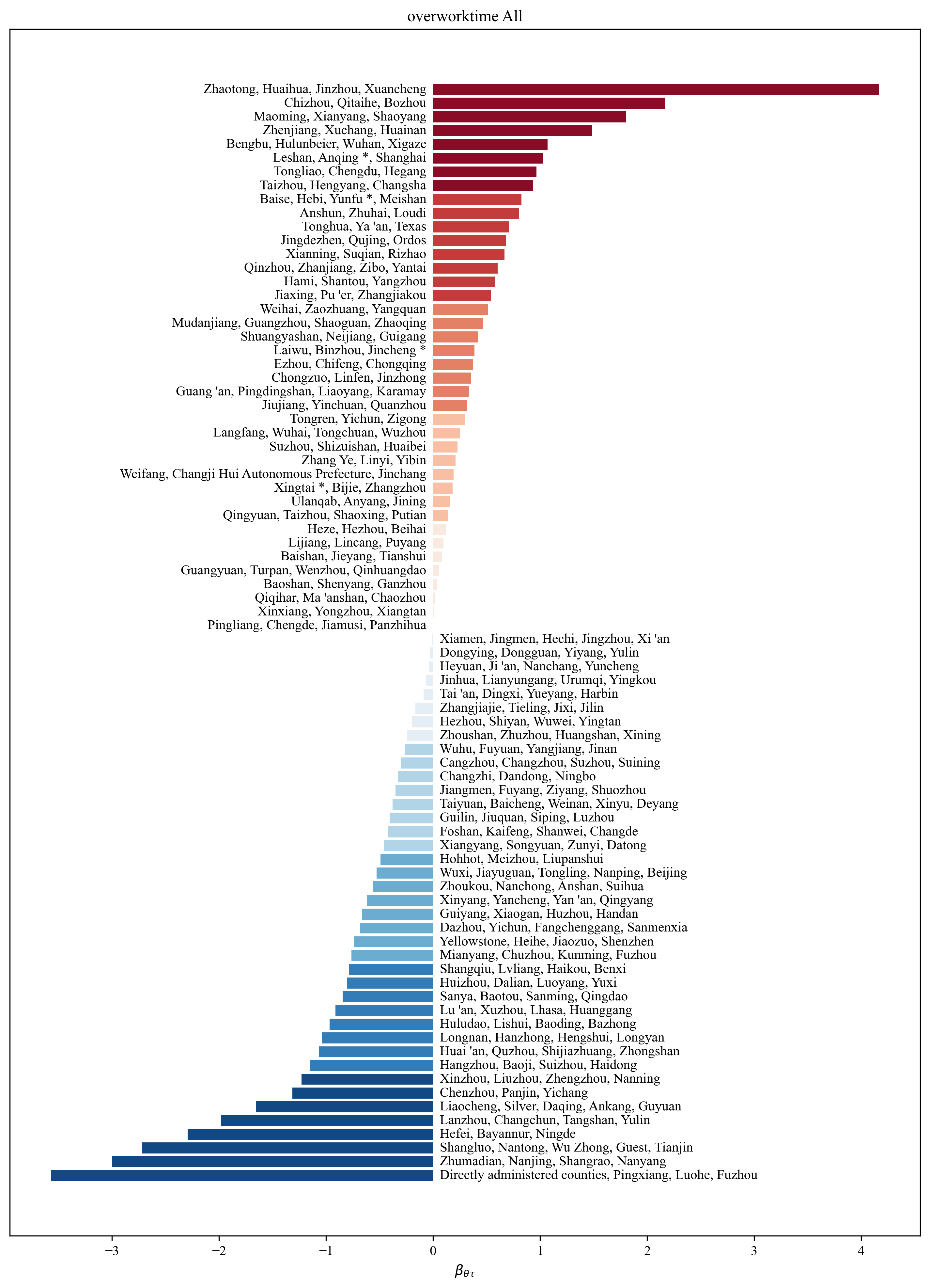


Fig. 2a. QQR estimates of effects of accessibility on overwork time in each city (all)


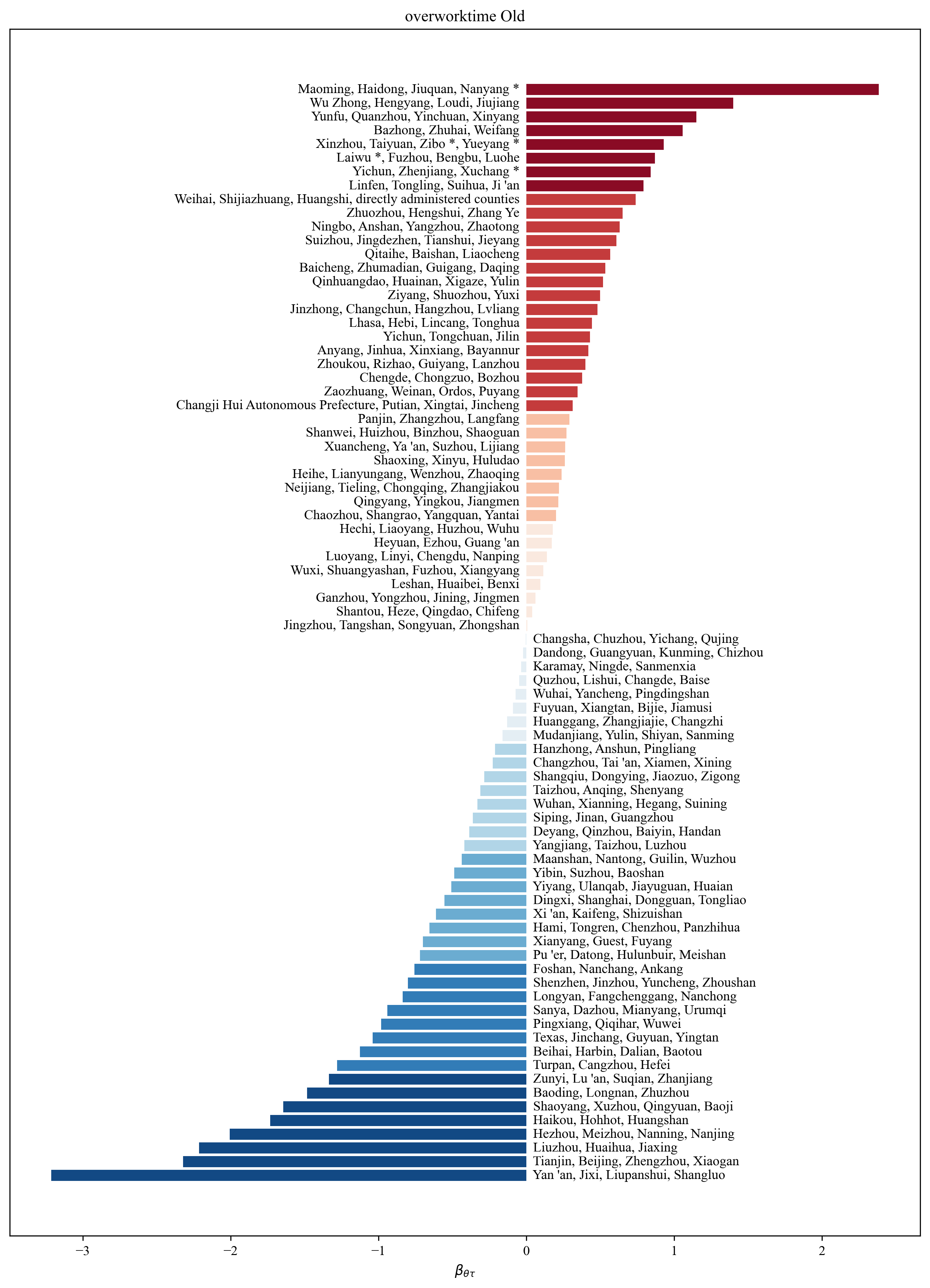


Fig. 2b. QQR estimates of effects of accessibility on overwork time in each city (first-generation)


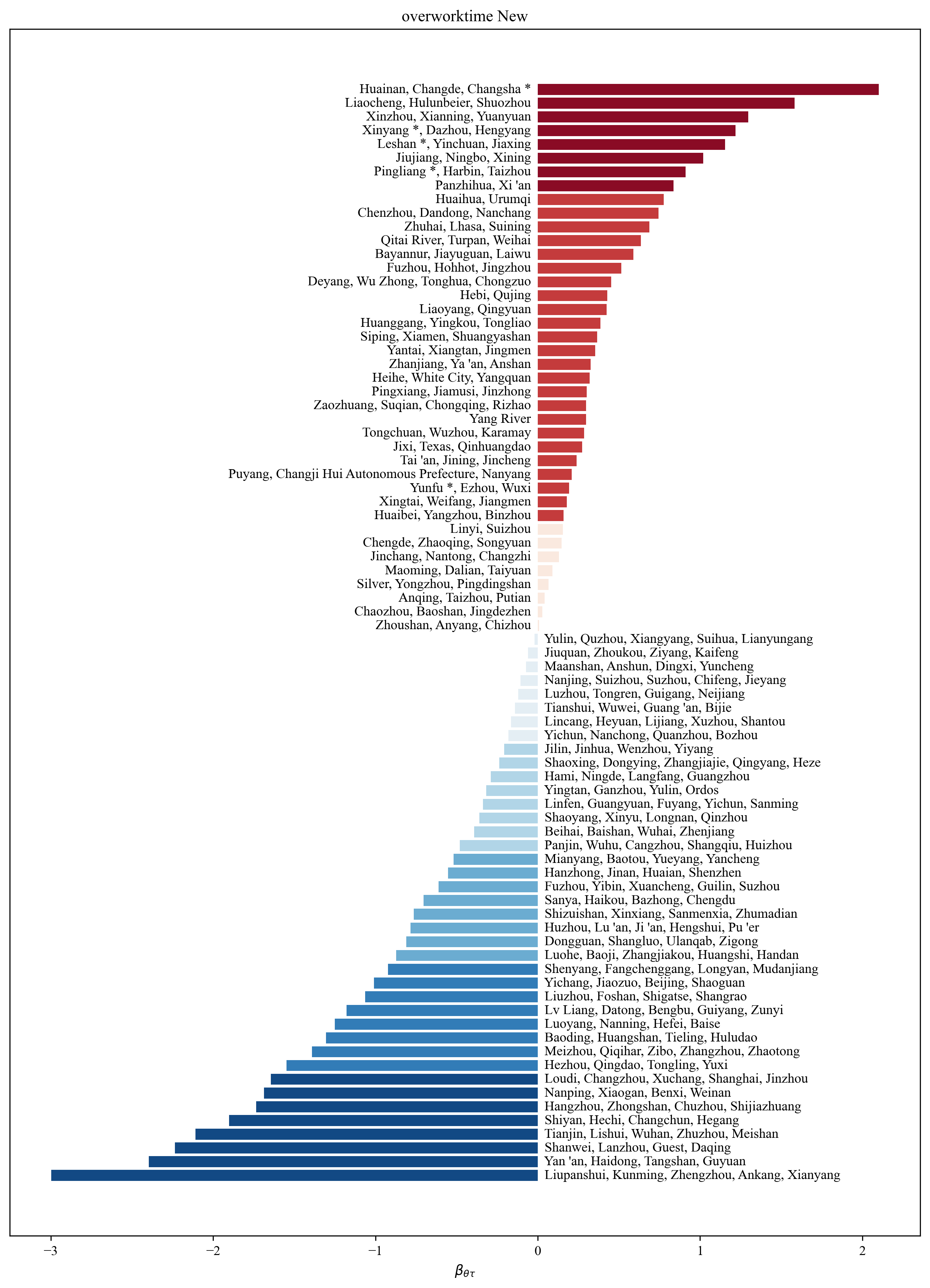


Fig. 2c. QQR estimates of effects of accessibility on overwork time in each city (new-generation)
